# Analytical Approach for a Heat Transfer Process through Nanofluid over an Irregular Porous Radially Moving Sheet by Employing KKL Correlation with Magnetic and Radiation Effects: Applications to Thermal System

**DOI:** 10.3390/mi13071109

**Published:** 2022-07-15

**Authors:** Umair Khan, Aurang Zaib, Anuar Ishak, Iskandar Waini, Zehba Raizah, Ahmed M. Galal

**Affiliations:** 1 Department of Mathematical Sciences, Faculty of Science and Technology, Universiti Kebangsaan Malaysia UKM, Bangi 43600, Malaysia; umairkhan@iba-suk.edu.pk (U.K.); anuar_mi@ukm.edu.my (A.I.); 2 Department of Mathematics and Social Sciences, Sukkur IBA University, Sukkur 65200, Pakistan; 3 Department of Mathematical Sciences, Federal Urdu University of Arts, Science & Technology, Gulshan-e-Iqbal, Karachi 75300, Pakistan; aurangzaib@fuuast.edu.pk; 4 Fakulti Teknologi Kejuruteraan Mekanikal dan Pembuatan, Universiti Teknikal Malaysia Melaka, Hang Tuah Jaya, Durian Tunggal 76100, Malaysia; iskandarwaini@utem.edu.my; 5 Department of Mathematics, College of Science, King Khalid University, Abha 62529, Saudi Arabia; 6 Mechanical Engineering Department, College of Engineering, Prince Sattam Bin Abdulaziz University, Wadi Addawaser 11991, Saudi Arabia; ahm.mohamed@psau.edu.sa; 7 Production Engineering and Mechanical Design Department, Faculty of Engineering, Mansoura University, P.O. Box 35516, Mansoura 35516, Egypt

**Keywords:** axisymmetric flow, nanofluid, magnetic field, radiation effect, KKL correlation, moving sheet

## Abstract

The aluminum nanoparticle is adequate for power grid wiring, such as the distribution of local power and the transmission of aerial power lines, because of its higher conductivity. This nanoparticle is also one of the most commonly used materials in applications in the electrical field. Thus, in this study, a radiative axisymmetric flow of Casson fluid, induced by water-based Al_2_O_3_ nanofluid by using the Koo–Kleinstreuer–Li (KKL) correlation, is investigated. The impact of the magnetic field is also taken into account. KKL correlation is utilized to compute the thermal conductivity and effective viscosity. Analytical double solutions are presented for the considered axisymmetric flow model after implementing the similarity technique to transmute the leading equations into ordinary differential equations. The obtained analytic forms were used to examine and discuss the velocity profile, the temperature distribution, reduced heat transfer, and coefficient of reduced skin friction. The analytic solutions indicate that the velocity profile decreases in the branch of the first solution and uplifts in the branch of the second solution due to the presence of an aluminum particle, whereas the dimensionless temperature enhances in both solutions. In addition, the Casson parameter increases the friction factor, as well as the heat transport rate.

## 1. Introduction

Heat transfer, which is regarded as the most important factor in most industrial and mechanical applications, is hampered by the profoundly low thermal conductivity of regular fluids. Heat transfer has a wide range of industrial applications that deal with both decreasing and increasing temperatures. Regular fluids, such as oil and water, have low thermal conductivity, which is the primary cause of engineering device inadequacy. In addition, the physical phenomena and parameters that influence the characteristics of the fluid flow and heat transfer in thermal systems composed of moving surfaces and working with nanofluids must be understood for the performance of these systems. Additionally, the fluid flow features, along with heat transfer in thermal systems, spanning from the automotive radiator to the nuclear reactor, must be analyzed to evaluate the performance of the system. Heat transfer can be improved to improve the thermal system performance. Steve Papell of NASA USA (NASA Washington D.C., United States, NASA Johnson Space Center) developed the term ferrofluids in 1963 to create an appropriate fluid for rocket fuel that could be drawn through an external magnetic field towards the pump inlet in a zero-gravity environment. Later on, Choi [1] utilized the nanofluid, which refers to a mixture formed by dispersing nanoparticles in a host fluid. He defined nanotechnology as heat transfer fluids that have better thermal properties than base fluids or regular fluids. Nanofluids are made up of small amounts of congested particles that are 100 nanometers or smaller in size. Hamad et al. [2] considered the viscous flow induced by nanofluid absorbed in a porous media. The impact of the convective condition on fluid flow and heat transport conveying nanofluid past a stretchable sheet was examined by Makinde and Aziz [3]. Rana and Bhargava [4] scrutinized the behavior of nanofluid flow subject to the elongating of a non-linear horizontal stretchable sheet. Pal and Mandal [5] examined the impact of VD (viscous dissipation) on radiative flow, conveying nanofluid past a stretchable/shrinking sheet saturated in a porous media. The variable temperature flux of nanofluid near a stagnation point through a thin horizontal needle was studied by Hayat et al. [6]. Eid [7] inspected the collective influence of the chemical reaction on mixed convective MHD (magnetohydrodynamics) flow encompassing a two-phase nanofluid model past an exponentially elongated sheet with heat generation. Zaib et al. [8] examined the effect of nanofluid on bio-convective flow past a heated wedge in a Darcy–Brinkman medium. The effect of a slip stimulus on axisymmetric fluid flowing past a shrinking cylinder induced by a water-based copper nanofluid with mixed convection was explored by Soomro et al. [9]. Khan et al. [10] scrutinized the concave and convex effects on radiative mixed convective flow through an erratic moving sheet induced by a hybrid nanofluid. Zaher et al. [11] examined the EOF (electro-osmotic forces) through the boundary layer flow induced by Williamson nanofluid with microorganisms in a non-Darcy medium. Gowda et al. [12] analyzed a 2D ferromagnetic flow of nanofluid induced by a magnetic dipole past an elastic flat sheet with the Stefan blowing condition. Recently, Shahid et al. [13] inspected the bi-convection flow induced by Carreau nanofluid past a paraboloid permeable upper surface with a chemical reaction and activation energy. 

The rheological properties of non-Newtonian fluids sparked widespread curiosity due to their prospective applications in an extensive range of technological, industrial, and pharmaceutical applications, such as crystal manufacturing and the manufacture offiber equipment. Non-Newtonian fluids are classified into numerous types, which are labeled as integral form (a sort of intensity), and differential form, for a broader understanding. Foams, melts, polymer solutions, and suspensions are offered as common examples of the materials that, under the right conditions, exhibit shear-thickening, shear-thinning, time-dependent, viscoplastic, and viscoelastic behavior. The model of Casson fluid is widely used to explain non-Newtonian fluids and their behavior. Casson fluid is identified as a shear-thinning fluid. It is a compelling fluid model because of its significant valuable inferences in our daily lives, such as in the polymer processing and biomedical fields. It offers a great framework for analyzing the two features in practice, apparent yield stress and Cason viscosity. Mukhopadhyay et al. [14] explored the two-dimensional flow of Casson fluid past an irregular stretchable sheet. Nadeem et al. [15] and Pramanik [16] scrutinized the Casson fluid flow past exponentially shrinking and stretching sheets, respectively. Haq et al. [17] examined the Casson fluid flow through a shrinkable heated sheet and found double solutions. Makinde et al. [18] incorporated the consequence of the MHD field on Casson fluid flow past a thermally horizontal melting stratified surface. Faraz et al. [19] observed the Soret/Dufour effects and chemical reactions on unsteady axisymmetric flow induced by Casson fluid past a radially stretchable sheet with multi-slip effects. The impact of the Lorentz force on non-linear radiative flow via a Casson fluid from a stretchable rotating heated disc was inspected by Khan et al. [20]. Zaib et al. [21] investigated the slip impacts on Casson fluid flow past a vertical plate immersed in Darcy–Brinkman medium with mixed convection and presented double solutions for slip parameters. Recently, Jyothi et al. [22] utilized the model of modified Buongiorno to investigate the effect of activation energy on the flow of Casson fluid induced by hybrid nanofluid past a downward/upward-moving rotatable disk. 

Consideration of the radiation effect adds a novel dimension to boundary-layer flow (BLF) and heat transport. The effects of radiative heat transport on dissimilar flows are critical in processes of high temperature and space technology. Thermal radiation can have a significant impact on heat transport and distribution of temperature in the BLF of a participating liquid at high temperatures. In recent years, significant endeavors were composed to obtain a better grip on the cooling rate. Some significant applications of radiative heat transfer include high-temperature plasmas, liquid metal fluids, cooling of a nuclear reactor, and systems of power generation. Several authors [23,24,25,26,27] studied distinct problems by considering the radiation effect on heat transfer. Daniel et al. [28] investigated unsteady radiative flow via a stretched sheet induced by nanofluid with the MHD field. Mekheimer et al. [29] inspected the influence of a chemical reaction on the radiative flow through Prandtl fluid with microorganisms past a porous, stretchable/shrinkable sheet. The impacts of radiation and melting on magneto Casson fluid via a porous, stretchable sheet with a chemical reaction were inspected numerically by Ramana et al. [30]. Abbas et al. [31] studied the impact of radiation on the squeezed flow through the suspension of particle fluid between parallel plates with oscillations. Lately, Khan et al. [32] discussed the radiation impact on blood flow induced by Casson fluid conveying gold particles through stretchable/shrinkable sheets with entropy generation.

The axisymmetric flow past a radially shrinking or stretching sheet has numerous manufacturing and industrial applications, including the production of glass fibers, mechanized plastic sheets and foods, polymer sheets, and coating for wires. According to a review of the literature, Arial [33] was the first to address axisymmetric flow over a linearly stretched sheet and to arrive at an exact and numerical solution. By using the variational iterative technique, Mirgolbabaei et al. [34] were able to provide an analytical solution to this problem. Ariel [35] expanded the axisymmetric flow problem to second-grade fluids. Sahoo [36] explored the role of partial slip on the magneto axisymmetric flow of viscoelastic fluid via a stretchable surface. Recently, Shahzad et al. [37] considered the axisymmetric flow through an exponential stretchable sheet with the magnetic field.

Based on the above literature reviews, it is apparent that, to the finest of the writer’s awareness, there is a lot of research and modeling about nanofluid flow through various surfaces in the literature. However, there are no results/efforts conducted with regard to the 2D flow stream of nanofluid past a porous, radially shrinkable sheet induced by Casson fluid with radiation effects using the KKL model. Thus, in this exploration, exact solutions of Casson fluid are presented by employing the KKL model correlation induced by Al_2_O_3_ nanofluid past a porous, radially shrinking sheet with radiation effect. In addition, the fluctuations in the velocity and thermal gradients are graphically deliberated.

## 2. Mathematical Modeling

### 2.1. Constitutive Equation

The state of the rheological equation for Casson incompressible fluid is stated as [38,39]

(1) $$\Gamma_{ij}=\{\begin{array}{c} 2\left(\mu_{b}+\frac{p_{y}}{\sqrt{2\pi }}\right)e_{ij},\pi >\pi_{c}\\ 2\left(\mu_{b}+\frac{p_{y}}{\sqrt{2\pi }}\right)e_{ij},\pi <\pi_{c} \end{array}.$$

where, $\pi =e_{ij}e_{ij}$ and $e_{ij}$ are the (i,j)th deformation rates’ components, π is signified as the production of deformation rates’ components with itself, $\pi_{c}$ is presented as critical value and $p_{y},\mu_{b}$ are identified as the yield stress and dynamic viscosity of the non-Newtonian fluid, respectively.

### 2.2. Basic Equations

**Figure 1:**
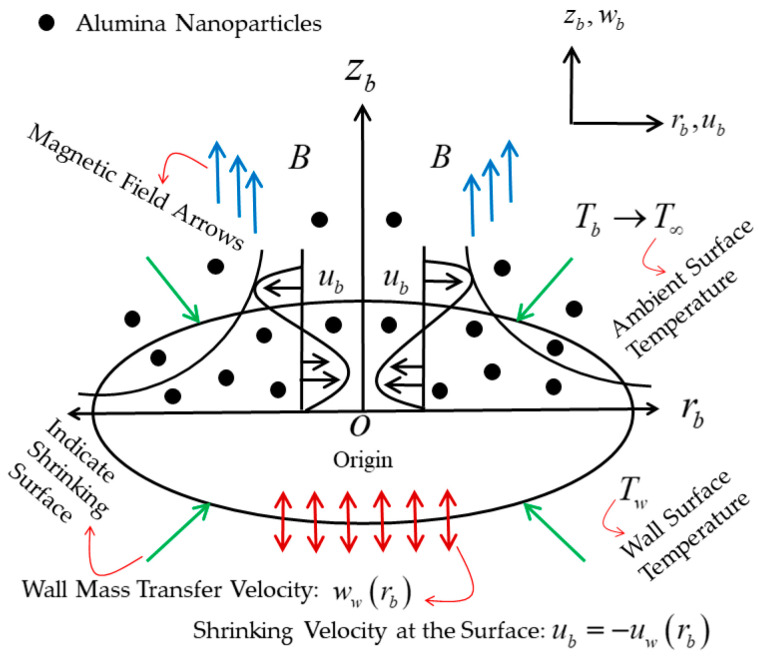
The physical model of the flow and coordinate system.

In the considered problem, the radiative axisymmetric flow and heat transport simulated by the (water-Al_2_O_3_) Casson nanofluid are investigated from a continuously non-linear, radially shrinking permeable sheet with variable wall temperature, comprising the correlation of the Koo–Kleinstreuer and Li (KKL) model. Therefore, the flow problem configuration is schematically revealed in Figure 1, where the Cartesian cylindrical coordinates $\left(r_{b},\delta ,z_{b}\right)$ are taken to be in such a way that the $r_{b}-$axis is considered parallel to the sheet and the $z_{b}-$axis is measured perpendicular to it, and the flow is occurring in the $\left(r_{b},\delta \right)-$plane. The considered flow is symmetric as well as axisymmetric about the $\left(r_{b},\delta \right)-$plane and $z_{b}-$axis, respectively. Meanwhile, the partial modification in all variables in relation to δ is going to be completely terminated, and mathematically, it is expressed as ∂/∂δ=0. In addition, the surface velocity is presumed as $u_{w}\left(r_{b}\right)=cr_{b}{}^{3}$, where c signifies as an arbitrary constant. Moreover, the exterior magnetic field is considered in changeable form as $B\left(r_{b}\right)=r_{b}B_{0}$, which carries out normally to the sheet’s surface (see Appendix A for derivation of the magnetic field). Furthermore, the sheet’s surface is also assumed to be permeable, and the corresponding wall mass transfer velocity is captured as $w_{w}\left(r_{b}\right)=-r_{b}v_{0}$, with $w_{w}<0$ and $v_{0}>0$ referring to mass blowing or injection, while $w_{w}>0$ and $v_{0}<0$ denote a mass suction phenomenon, respectively. Additionally, the temperature is considered as $T_{\infty }<T_{w}$, where $T_{\infty }$ and $T_{w}$ designate the steady freestream temperature and the variable or power law wall temperature, respectively.

The properties of the aluminum oxide (Al_2_O_3_) nanoparticles and the base fluid (H_2_O) are taken to be constant and no slip happens between them. The nanofluid thermophysical features are specified in Table 1. Under these postulations, the steady basic governing equations in the partial differential equations form are written as follows [40]:

(2) $$\frac{\partial u_{b}}{\partial r_{b}}+\frac{u_{b}}{r_{b}}+\frac{\partial w_{b}}{\partial z_{b}}=0,$$

(3) $$u_{b}\frac{\partial u_{b}}{\partial r_{b}}+w_{b}\frac{\partial u_{b}}{\partial z_{b}}=\frac{\mu_{\mathrm{nf}}}{\rho_{\mathrm{nf}}}\left[\frac{\left(\chi +1\right)}{\chi }\right]\frac{\partial^{2}u_{b}}{\partial z_{b}^{2}}-\sigma_{\mathrm{nf}}\frac{B^{2}\left(r_{b}\right)}{\rho_{\mathrm{nf}}}u_{b},$$

(4) $$u_{b}\frac{\partial T_{b}}{\partial r_{b}}+w_{b}\frac{\partial T_{b}}{\partial z_{b}}=\frac{k_{\mathrm{nf}}}{{\left(\rho c_{p}\right)}_{\mathrm{nf}}}\frac{\partial^{2}T_{b}}{\partial z_{b}^{2}}-\frac{1}{{\left(\rho c_{p}\right)}_{\mathrm{nf}}}\frac{\partial }{\partial z_{b}}\left(q_{\mathrm{rad}}\right),$$

where $w_{b}$ and $u_{b}$ symbolize the velocity components in the respective direction of the $z_{b}-$ and $r_{b}-$axes, respectively, $T_{b}$ is the temperature of the fluid and χ is the non-Newtonian or Casson fluid parameter, while the other mathematical letters are recognized and determined later for the nanofluid and KKL (Koo–Kleinstreuer and Li) model. The viscous dissipation (VD) term is ignored in the suggested equation of energy (4), whilst the radiative term of heat flux, $q_{\mathrm{rad}}$, is appended through the Rosseland approximations in the easiest form as follows:

(5) $$q_{rad}=\frac{-16\sigma_{C}T_{\infty }^{3}}{3k_{C}}\frac{\partial T_{b}}{\partial z_{b}}$$

Here, in the aforementioned Equation (5), $\sigma_{C}$ and $k_{C}$ delegate the constant of Stefan Boltzmann and the coefficient of mean absorption. For further simplicity, the Equation (4), together with Equation (5), can be preserved in a new compact form as:

(6) $$u_{b}\frac{\partial T_{b}}{\partial r_{b}}+w_{b}\frac{\partial T_{b}}{\partial z_{b}}=\frac{k_{f}}{{\left(\rho c_{p}\right)}_{\mathrm{nf}}}\left(\frac{k_{nf}}{k_{f}}+\frac{4}{3}R_{d}\right)\frac{\partial^{2}T_{b}}{\partial z_{b}^{2}},$$

where $k_{f}$ represents the base fluid’s electrical conductivity and $R_{d}=\frac{4\sigma_{C}T_{\infty }^{3}}{k_{C}k_{f}}$ represents the dimensionless radiation parameter. The BCs (boundary conditions) of the problem are:

(7) $$\begin{array}{l} \;\;u_{b}=-u_{w}\left(r_{b}\right),\;w_{b}=w_{w}\left(r_{b}\right),\;T_{b}=T_{w}\;\left(T_{w}=T_{\infty }+r_{b}^{m}T_{0}\right)\mathrm{at}\;z_{b}=0,\\ \;\;\;\;\;\;\;\;\;\;\;\;\;u_{b}\to 0,T_{b}\to T_{\infty }\;\;\;\mathrm{as}\;\;\;z_{b}\to \infty , \end{array}\}$$

where $T_{0}$ is the constant reference temperature, and m is the relative factor of the power law index. Furthermore, the rest of the mathematical notations in the governing equation are namely illustrated as ${\left(\rho c_{p}\right)}_{\mathrm{nf}}$ the effective specific heat capacity at the constant pressure, $\sigma_{\mathrm{nf}}$ the effective electrical conductivity, and $\rho_{\mathrm{nf}}$ the effective density of the nanofluid, which is demarcated as (see [41,42]):

(8) $$\begin{array}{l} {\left(\rho c_{p}\right)}_{\mathrm{nf}}=\phi {\left(\rho c_{p}\right)}_{\mathrm{pa}}+\left(1-\phi \right){\left(\rho c_{p}\right)}_{f},\rho_{\mathrm{nf}}=\phi \rho_{\mathrm{pa}}+\left(1-\phi \right)\rho_{f},\\ \sigma_{\mathrm{nf}}=\left[1+\frac{3\left(\sigma_{\mathrm{pa}}/\sigma_{f}-1\right)\phi }{\left(\sigma_{\mathrm{pa}}/\sigma_{f}+2\right)-\left(\sigma_{\mathrm{pa}}/\sigma_{f}-1\right)\phi }\right]\sigma_{f}, \end{array}$$

where ϕ signifies the solid nanoparticle volume fraction. Additionally, ${\left(\rho c_{p}\right)}_{f},\sigma_{f}$, and $\rho_{f}$ are the respective quantities of the regular-based fluid. However, the subscripts in Equation (8) are the following: f,pa and nf, which reveal the regular-based fluid, nanoparticles, and nanofluid, respectively. 

The Brownian motion has a substantial effect on the thermal conductivity, $k_{\mathrm{nf}}$, of the nanofluid. Koo and Kleinstreuer [43] suggested that $k_{\mathrm{nf}}$ consists of the conventional static part of the particle and the Brownian part of the motion. The considered model of $k_{\mathrm{nf}}$ consists of two components that take the impacts of temperature dependence, the volume fraction of particle and particle size, and types of particle and fluid base groupings into account. Let us mathematically express it as [43]:

(9) $$k_{\mathrm{nf}}=k_{\mathrm{Brownian}}+k_{\mathrm{static}}$$

(10) $$\frac{k_{\mathrm{static}}}{k_{f}}=1+\frac{3\left(\frac{k_{\mathrm{pa}}}{k_{f}}-1\right)\phi }{\left(\frac{k_{\mathrm{pa}}}{k_{f}}+2\right)-\left(\frac{k_{\mathrm{pa}}}{k_{f}}-1\right)\phi }$$

where $k_{\mathrm{static}}$ signifies the Maxwell classical correlation-based static thermal conductivity. A simulation of the flow of Stokes around a sphere results in an enriched $k_{f}$, produced by a convective micro-size particle and a heat transfer of Brownian particle speed, affecting the far-field fluid movement (nanomaterials). Additionally, to the influence of temperature’s role in the given model, the interaction between nanoparticles was combined by the introduction of two empirical functions (γ and h) Koo [44], which leads to:

(11) $$k_{\mathrm{Brownian}}=5\times 10^{4}\gamma \phi \rho_{f}c_{p,f}\sqrt{\frac{\kappa_{b}T_{b}}{\rho_{\mathrm{pa}}d_{\mathrm{pa}}}}h\left(T_{b},\phi \right)$$

The significance of the interfacial thermal resistance between base fluid and nanoparticles was emphasized progressively in recent years (see, Jang and Choi [45] and Prasher et al. [46]). In the nearby layers of two dissimilar types of material, such as the TIR, Kapitza resistance is alleged to be present; the thin barrier layer participates a crucial role in deteriorating the effective thermal conductivity of the nanoparticles. Li [47] reviewed the Koo and Kleinstreuer model [43] and combined γ and h functions to build up a new *H* function to capture the impacts of temperature, particle diameter, and nanoparticles on volume fraction. The empirical (*H*-function) of the nanofluid depends upon its type [48]. In addition, the original $\left(k_{\mathrm{pa}}\right)$ in Equation (10) was changed by the new $\left(k_{\mathrm{pa},\;\mathrm{eff}}\right)$ form (given below) with the introduction of the thermal interfacial resistance ($R_{f}=4\times 10^{-8}\;km^{3}/W$):

(12) $$R_{f}+\frac{d_{\mathrm{pa}}}{k_{\mathrm{pa}}}=\frac{d_{\mathrm{pa}}}{k_{\mathrm{pa},\;\mathrm{eff}}}$$

The function should be dissimilar for distinct nanoparticles and regular fluids. The current study only considers water-based nanofluids. For water-Al_2_O_3_ nanofluids, the following format should be read as follows:

(13) $$\begin{array}{l} H\left(T_{b},\phi ,d_{\mathrm{pa}}\right)=\left(b_{1}+b_{2}\ln\left(d_{\mathrm{pa}}\right)+b_{5}\ln{\left(d_{\mathrm{pa}}\right)}^{2}+b_{3}\ln\left(\phi \right)+b_{4}\ln\left(d_{\mathrm{pa}}\right)\ln\left(\phi \right)\right)\ln\left(T_{b}\right)+\\ \left(b_{6}+b_{7}\ln\left(d_{\mathrm{pa}}\right)+b_{10}\ln{\left(d_{\mathrm{pa}}\right)}^{2}+b_{8}\ln\left(\phi \right)+b_{9}\ln\left(d_{\mathrm{pa}}\right)\ln\left(\phi \right)\right),\\ \phi \leq 0.04,\;\;\;\;\;\;\;\;\;\;\;\;\;\;\;\;\;\;\;\;\;\;300K\leq T_{b}\leq 325K. \end{array}$$

The $R^{2}$ of water- Al_2_O_3_ nanofluid is 96% and 98%, respectively [48] (Table 2). The coefficients $b_{i}\;(i=1\ldots 10)$ are based on the type of nanoparticles, and also on these coefficients. Finally, the correlation of KKL (Koo–Kleinstreuer–Li) is given by:

(14) $$k_{\mathrm{Brownian}}=5\times 10^{4}\phi \rho_{f}c_{p,f}\sqrt{\frac{\kappa_{b}T_{b}}{\rho_{\mathrm{pa}}d_{\mathrm{pa}}}}H\left(T_{b},\phi ,d_{\mathrm{pa}}\right)$$

Koo and Kleinstreuer [43] examined the laminar nanofluid flow in micro-heat sinks using their efficient thermal conductivity model of nanofluid. Therefore, in regard to the absolute $\mu_{\mathrm{nf}}$ (viscosity) owing to the suspension of micro-mixing, the proposed expression is stated as:

(15) $$\mu_{nf}=\mu_{\mathrm{Brownian}}+\mu_{\mathrm{static}}=\mu_{\mathrm{static}}+\frac{\mu_{f}k_{\mathrm{Brownian}}}{\mathrm{Pr}k_{f}}$$

where $\mu_{\mathrm{static}}=\frac{\mu_{f}}{{\left(1-\phi \right)}^{2.5}}$ represents a nanofluid static viscosity that Brinkman originally defines.

### 2.3. Non-Dimensional Equations

To facilitate the investigation of the problem, the appropriate self-similarity variables are proposed here:

(16) $$\xi =\sqrt{\frac{c}{\nu_{f}}}z_{b}r_{b},\theta \left(\xi \right)=\frac{T-T_{\infty }}{T_{w}-T_{\infty }},\;\psi =-\sqrt{c\nu_{f}}r_{b}^{3}G\left(\xi \right)$$

where $\nu_{f}$ is the kinematic viscosity and ψ is the stream function. Next, employ the description of ψ in component form as $u_{b}=\frac{1}{r_{b}}\frac{\partial \psi }{\partial z_{b}}$ and $w_{b}=-\frac{1}{r_{b}}\frac{\partial \psi }{\partial r_{b}}$. Now, utilize Equation (16) in these given equations to acquire the velocities in the following form:

(17) $$u_{b}={\mathrm{cr}}_{b}^{3}G\prime \left(\xi \right)\;\;\mathrm{and}\;w_{b}=-\sqrt{c\nu_{f}}\;r_{b}\left(3G\left(\xi \right)+\xi G\prime \left(\xi \right)\right)$$

Thus, the similarity transformation and, the equation of continuity is held, whilst the leading Equations (3) and (6), along with the BCs (7), are eased to the dimensional form of ODEs as follows:

(18) $$\frac{\mu_{\mathrm{nf}}/\mu_{f}}{\rho_{\mathrm{nf}}/\rho_{f}}\left[\frac{\left(\chi +1\right)}{\chi }\right]G\prime \prime \prime +3GG\prime \prime -3G\prime^{2}-\frac{\sigma_{\mathrm{nf}}/\sigma_{f}}{\rho_{\mathrm{nf}}/\rho_{f}}M_{b}G\prime =0,$$

(19) $$\frac{1}{\mathrm{Pr}{\left(\rho c_{p}\right)}_{\mathrm{nf}}/{\left(\rho c_{p}\right)}_{f}}\left(k_{\mathrm{nf}}/k_{f}+\frac{4}{3}R_{d}\right)\theta \prime \prime +3G\theta \prime -m\theta G\prime =0,$$

subject to the following BCs:

(20) $$\begin{array}{l} G\prime \left(\xi \right)=-1,G\left(\xi \right)=S_{b},\;\;\theta \left(\xi \right)=1\;\;\mathrm{at}\;\;\xi =0\\ G\prime \left(\xi \right)\to 0,\;\;\theta \left(\xi \right)\to 0\;\;\mathrm{as}\;\;\xi \to \infty \end{array}\}$$

where primes (′) exemplify the differentiation w.r.t ξ. The dimensionless constants involved in the problem are the magnetic parameter $M_{b}$ and the mass transfer factor $S_{b}$, with $S_{b}>0$ for suction and $S_{b}<0$ for injection, posited mathematically as follows:

(21) $$M_{b}=\frac{\sigma_{f}B_{0}^{2}}{\rho_{f}c},S_{b}=\frac{v_{0}}{3\sqrt{c\nu_{f}}}$$

### 2.4. Gradients

The wall drag force or shear stress $C_{g}$ and heat transfer $Nu_{r_{b}}$ are important gradients for engineering physical quantities of attention that are used for the behavior of the fluid flow dynamics, as well as the local heat transfer characteristics. These gradients are mathematically defined as

(22) $$C_{g}=\frac{2\mu_{\mathrm{nf}}}{\rho_{f}u_{w}^{2}}{\left(\frac{\partial u_{b}}{\partial z_{b}}\right)|}_{z_{b}=0},\;\;\;\;\;\;\;\;\;\;\;Nu_{r_{b}}=-\frac{r_{b}k_{\mathrm{nf}}}{k_{f}\left(T_{w}-T_{\infty }\right)}{\left(\frac{\partial T_{b}}{\partial z_{b}}\right)|}_{z_{b}=0}.$$

Replacing Equation (16) into the aforementioned Equation (22), we find the required reduced form of the wall drag force and heat transfer, which can take place as follows:

(23) 12Rerb1/2Cg=μnfμfG″(0),           Rerb−1/2Nurb=−knfkfθ′(0),

where Rerb=uw(rb)rbνf signifies the Reynolds number.

## 3. Analytic Solutions Methodology

This section of the paper illustrates how the analytical solution technique works. In this considered problem, we must determine the closed-form precise solutions to Equations (18) and (19) subject to the required BCs (20). Therefore, keeping the comparable previous papers approach in mind [49,50,51], we acquire the analytical or exact solution of Equation (18), subject to BCs (20) in the following form:

(24) $$G\left(\xi \right)=S_{b}-\frac{1}{D}\left(1-e^{-D\xi }\right).$$

Plugging Equation (24) for dimensionless similarity in Equation (18) yields the following second-order algebraic equation, which may be interpreted as:

(25) $$\frac{\mu_{nf}}{\mu_{f}}\left[\frac{\left(\chi +1\right)}{\chi }\right]D^{2}-3S_{b}\frac{\rho_{nf}}{\rho_{f}}D+3\frac{\rho_{nf}}{\rho_{f}}-\frac{\sigma_{nf}}{\sigma_{f}}M_{b}=0$$

where

$$D=\frac{3S_{b}\left(\rho_{nf}/\rho_{f}\right)\pm \sqrt{9S_{b}^{2}{\left(\rho_{nf}/\rho_{f}\right)}^{2}-4\left(\mu_{nf}/\mu_{f}\right)\left[\frac{\left(\chi +1\right)}{\chi }\right]\left(3\left(\rho_{nf}/\rho_{f}\right)-\left(\sigma_{nf}/\sigma_{f}\right)M_{b}\right)}}{2\left(\mu_{nf}/\mu_{f}\right)\left[\frac{\left(\chi +1\right)}{\chi }\right]}$$

Furthermore, the suggested problem has upper and lower branch solutions (dual or multiple solutions), as evidenced by the two distinct roots mentioned above. Additionally, Equation (24) depicts the velocity G′(ξ) and shear stress G″(ξ) at the wall surface, which are given by:

(26) $$G\prime \left(\xi \right)=-e^{-D\xi }$$

and

(27) $$G\prime \prime \left(0\right)=\frac{3S_{b}\left(\rho_{nf}/\rho_{f}\right)\pm \sqrt{9S_{b}^{2}{\left(\rho_{nf}/\rho_{f}\right)}^{2}-4\left(\mu_{nf}/\mu_{f}\right)\left[\frac{\left(\chi +1\right)}{\chi }\right]\left(3\left(\rho_{nf}/\rho_{f}\right)-\left(\sigma_{nf}/\sigma_{f}\right)M_{b}\right)}}{2\left(\mu_{nf}/\mu_{f}\right)\left[\frac{\left(\chi +1\right)}{\chi }\right]}$$

To decipher Equation (19) exactly with the BC (20), we require the solution of Equation (24) in the realizable energy equation to catch the requisite form as follows:

(28) $$\theta \prime \prime +\frac{3\mathrm{Pr}{\left(\rho c_{p}\right)}_{nf}/{\left(\rho c_{p}\right)}_{f}}{\left(\frac{k_{nf}}{k_{f}}+\frac{4}{3}R_{d}\right)}\left(S_{b}-\frac{1}{D}+\frac{1}{D}e^{-D\xi }\right)\theta \prime +\frac{m\mathrm{Pr}{\left(\rho c_{p}\right)}_{nf}/{\left(\rho c_{p}\right)}_{f}}{\left(\frac{k_{nf}}{k_{f}}+\frac{4}{3}R_{d}\right)}\theta e^{-D\xi }=0$$

Here, we suppose a new variable $\eta =\frac{3\mathrm{Pr}{\left(\rho c_{p}\right)}_{nf}/{\left(\rho c_{p}\right)}_{f}}{\left(k_{nf}/k_{f}+\frac{4}{3}R_{d}\right)D^{2}}e^{-D\xi }$ for Equation (28) to find their closed-form analytical solution. Therefore, implementing a new variable in the Equation (28) and the border ailment (20) yields:

(29) $$\eta \frac{d^{2}\theta }{d\eta^{2}}+\left(L-\eta \right)\frac{d\theta }{d\eta }-P\theta =0,$$

where $L=1-\frac{3\mathrm{Pr}{\left(\rho c_{p}\right)}_{nf}/{\left(\rho c_{p}\right)}_{f}}{\left(\frac{k_{nf}}{k_{f}}+\frac{4}{3}R_{d}\right)D}\left(S_{b}-\frac{1}{D}\right)$ and $P=-\frac{m}{3}$.

In terms of a new variable, the subject BCs are:

(30) $$\theta \left(\frac{3\mathrm{Pr}{\left(\rho c_{p}\right)}_{nf}/{\left(\rho c_{p}\right)}_{f}}{\left(\frac{k_{nf}}{k_{f}}+\frac{4}{3}R_{d}\right)D^{2}}\right)=1\;\;\;\mathrm{and}\;\theta \left(0\right)=0$$

Thus, the general solution for Equation (29) is given as:

(31) $$\theta \left(\eta \right)=B_{1}M\left(P,L,\eta \right)+B_{2}\eta^{1-L}M\left(P+1-L,2-L,\eta \right)$$

where M is the first kind of confluent hypergeometric function or Kummer function with $B_{1}$ and $B_{2}$ being arbitrary constants.

Using the values of L and P along with BCs (30) in the general solution of Equation (31), we get:

(32) $$\theta \left(\eta \right)=\frac{{\left(\frac{\eta \left(k_{nf}/k_{f}+\frac{4}{3}R_{d}\right)D^{2}}{3\mathrm{Pr}{\left(\rho c_{p}\right)}_{nf}/{\left(\rho c_{p}\right)}_{f}}\right)}^{D_{b}}M\left(D_{b}-\frac{m}{3},1+D_{b},\eta \right)}{M\left(D_{b}-\frac{m}{3},1+D_{b},\frac{3\mathrm{Pr}{\left(\rho c_{p}\right)}_{nf}/{\left(\rho c_{p}\right)}_{f}}{\left(k_{nf}/k_{f}+\frac{4}{3}R_{d}\right)D^{2}}\right)}$$

$$D_{b}=\frac{3\mathrm{Pr}{\left(\rho c_{p}\right)}_{nf}/{\left(\rho c_{p}\right)}_{f}}{\left(k_{nf}/k_{f}+\frac{4}{3}R_{d}\right)D}\left(S_{b}-\frac{1}{D}\right)$$

Thus, the analytical solution of the temperature, in terms of ξ, is given by as:

(33) $$\theta \left(\xi \right)=\frac{e^{-DD_{b}\xi }M\left(D_{b}-\frac{m}{3},1+D_{b},\frac{3\mathrm{Pr}{\left(\rho c_{p}\right)}_{nf}/{\left(\rho c_{p}\right)}_{f}}{\left(k_{nf}/k_{f}+\frac{4}{3}R_{d}\right)D^{2}}e^{-D\xi }\right)}{M\left(D_{b}-\frac{m}{3},1+D_{b},\frac{3\mathrm{Pr}{\left(\rho c_{p}\right)}_{nf}/{\left(\rho c_{p}\right)}_{f}}{\left(k_{nf}/k_{f}+\frac{4}{3}R_{d}\right)D^{2}}\right)}$$

Now captivating the first derivative of Equation (32) w.r.t ξ, it becomes

(34) $$\begin{array}{l} \theta \prime \left(\xi \right)=-\frac{DD_{b}e^{-DD_{b}\xi }M\left(D_{b}-\frac{m}{3},1+D_{b},\frac{3\mathrm{Pr}{\left(\rho c_{p}\right)}_{nf}/{\left(\rho c_{p}\right)}_{f}}{\left(k_{nf}/k_{f}+\frac{4}{3}R_{d}\right)D^{2}}e^{-D\xi }\right)}{M\left(D_{b}-\frac{m}{3},1+D_{b},\frac{3\mathrm{Pr}{\left(\rho c_{p}\right)}_{nf}/{\left(\rho c_{p}\right)}_{f}}{\left(k_{nf}/k_{f}+\frac{4}{3}R_{d}\right)D^{2}}\right)}\\ -\frac{3\left(D_{b}-\frac{m}{3}\right)e^{-D\xi -D_{b}D\xi }\mathrm{Pr}{\left(\rho c_{p}\right)}_{nf}/{\left(\rho c_{p}\right)}_{f}M\left(D_{b}-\frac{m}{3}+1,2+D_{b},\frac{3\mathrm{Pr}{\left(\rho c_{p}\right)}_{nf}/{\left(\rho c_{p}\right)}_{f}}{\left(k_{nf}/k_{f}+\frac{4}{3}R_{d}\right)D^{2}}e^{-D\xi }\right)}{\left(k_{nf}/k_{f}+\frac{4}{3}R_{d}\right)D\left(1+D_{b}\right)M\left(D_{b}-\frac{m}{3},1+D_{b},\frac{3\mathrm{Pr}{\left(\rho c_{p}\right)}_{nf}/{\left(\rho c_{p}\right)}_{f}}{\left(k_{nf}/k_{f}+\frac{4}{3}R_{d}\right)D^{2}}\right)}. \end{array}$$

The heat transfer rate at the wall is specified as

(35) $$-\theta \prime \left(0\right)=DD_{b}+\frac{3\left(D_{b}-\frac{m}{3}\right)\mathrm{Pr}{\left(\rho c_{p}\right)}_{nf}/{\left(\rho c_{p}\right)}_{f}M\left(D_{b}-\frac{m}{3}+1,2+D_{b},\frac{3\mathrm{Pr}{\left(\rho c_{p}\right)}_{nf}/{\left(\rho c_{p}\right)}_{f}}{\left(k_{nf}/k_{f}+\frac{4}{3}R_{d}\right)D^{2}}\right)}{\left(k_{nf}/k_{f}+\frac{4}{3}R_{d}\right)D\left(1+D_{b}\right)M\left(D_{b}-\frac{m}{3},1+D_{b},\frac{3\mathrm{Pr}{\left(\rho c_{p}\right)}_{nf}/{\left(\rho c_{p}\right)}_{f}}{\left(k_{nf}/k_{f}+\frac{4}{3}R_{d}\right)D^{2}}\right)}.$$

## 4. Results and Discussion

**Figure 2:**
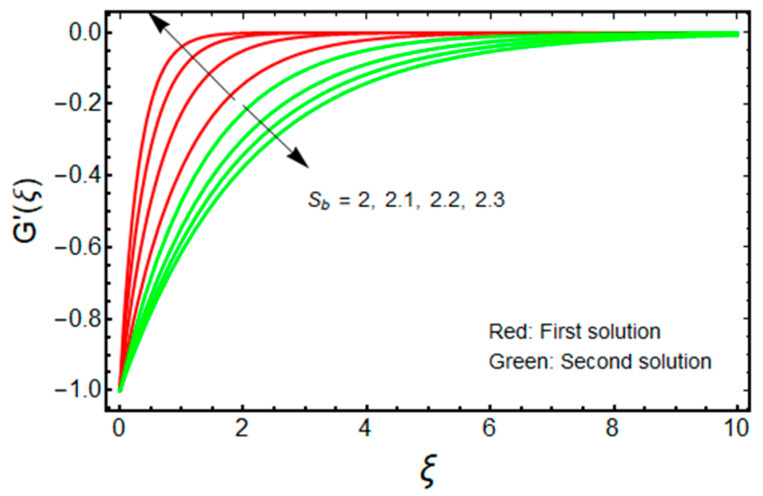
Effect of $S_{b}$ on G′(ξ) when $\phi =0.025,\;\chi =1,\;M_{b}=0.5$.

**Figure 3:**
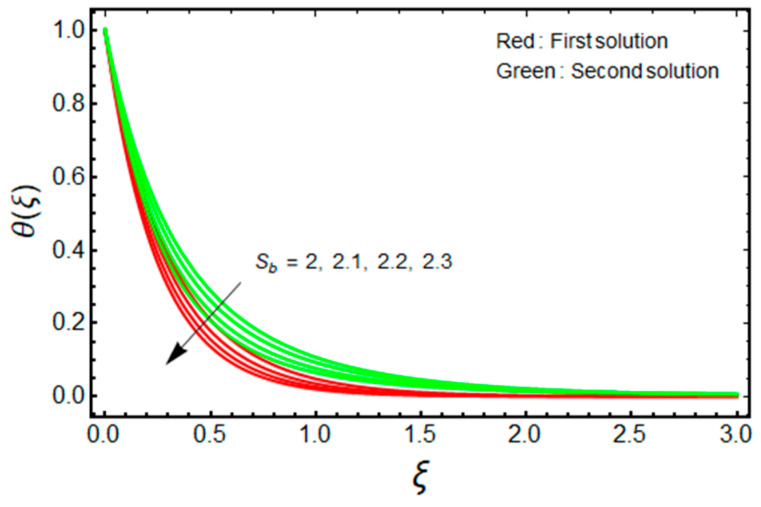
Effect of $S_{b}$ on θ(ξ) when $\phi =0.025,\;\chi =1,\;M_{b}=0.5,\;R_{d}=1.5,\;m=1$.

**Figure 4:**
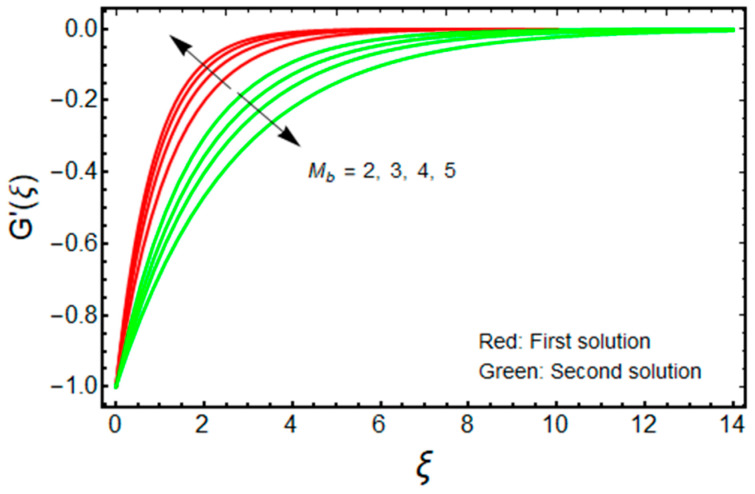
Effect of $M_{b}$ on G′(ξ) when $\phi =0.025,\;\chi =1,\;S_{b}=2.5$.

**Figure 5:**
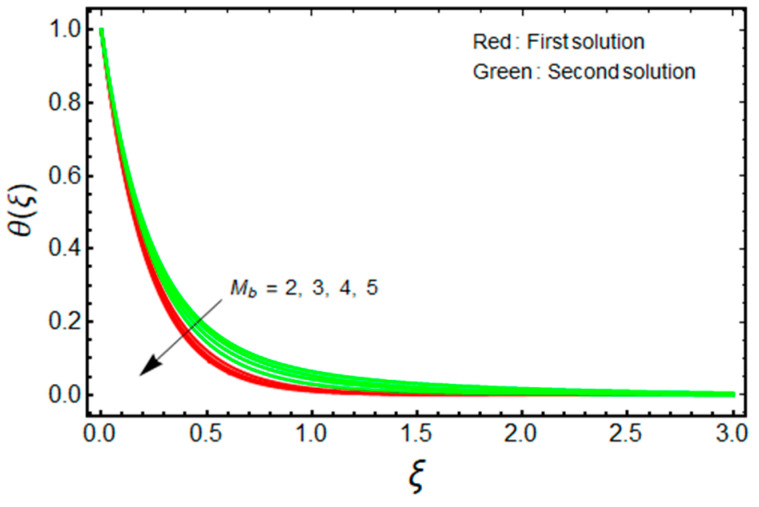
Effect of $M_{b}$ on θ(ξ) when $\phi =0.025,\;\chi =1,\;S_{b}=2.5,\;R_{d}=1.5,\;m=1$.

**Figure 6:**
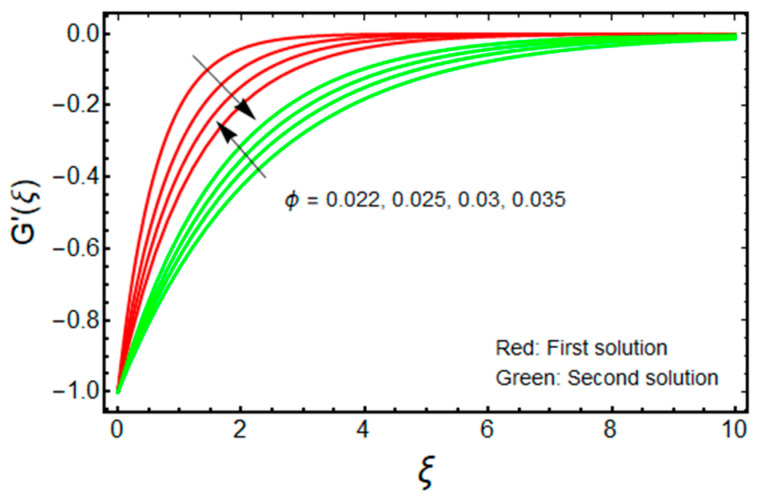
Effect of ϕ on G′(ξ) when $S_{b}=2.5,\;\chi =1,\;M_{b}=0.5$.

**Figure 7:**
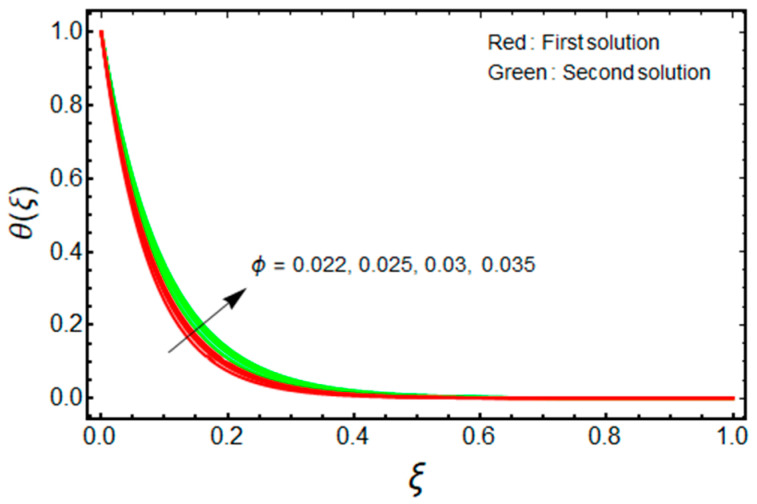
Effect of ϕ on θ(ξ) when $S_{b}=2.5,\;\chi =1,\;M_{b}=0.5,\;R_{d}=1.5,\;m=1$.

**Figure 8:**
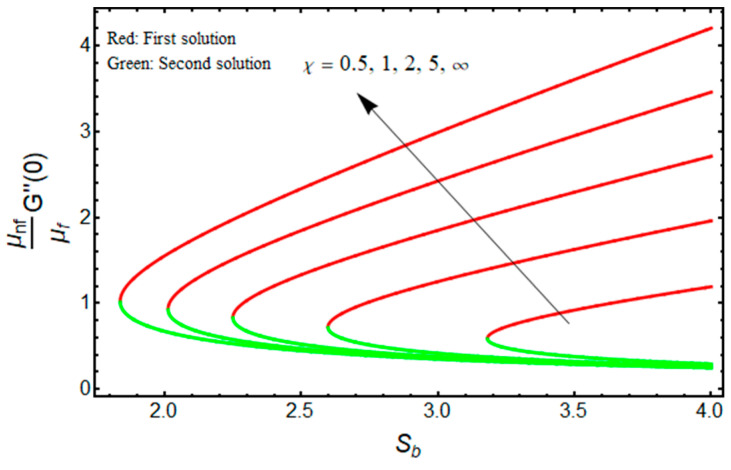
Effect of χ on $\left(\mu_{nf}/\mu_{f}\right)G\prime \prime \left(0\right)\;$ versus $S_{b}$ when $\phi =0.025,\;M_{b}=0.5$.

**Figure 9:**
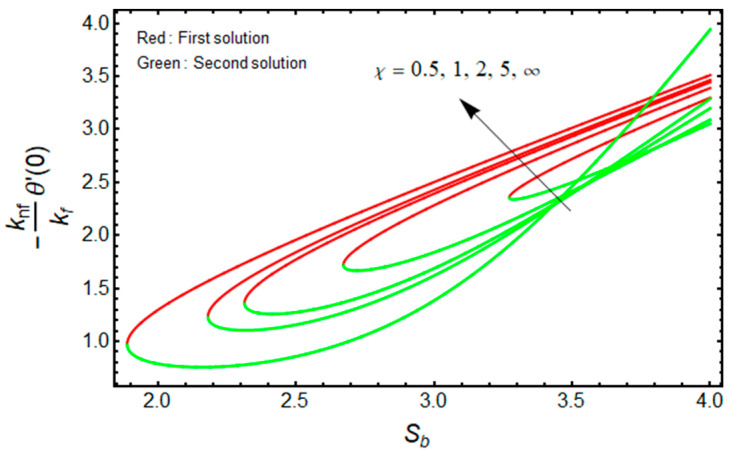
Effect of χ on $-\left(k_{nf}/k_{f}\right)\theta \prime \left(0\right)$ versus $S_{b}$ when $\phi =0.025,\;M_{b}=0.5,\;R_{d}=1.5,\;m=1$.

**Figure 10:**
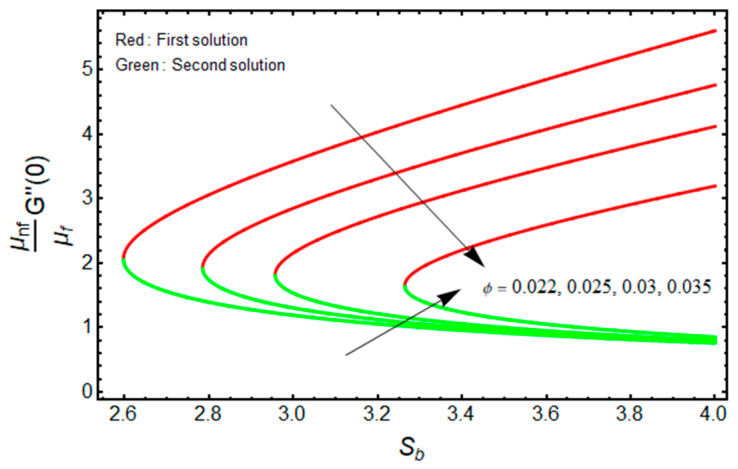
Effect of χ on $\left(\mu_{nf}/\mu_{f}\right)G\prime \prime \left(0\right)\;$ versus $S_{b}$ when $\chi =1,\;M_{b}=0.5$.

**Figure 11:**
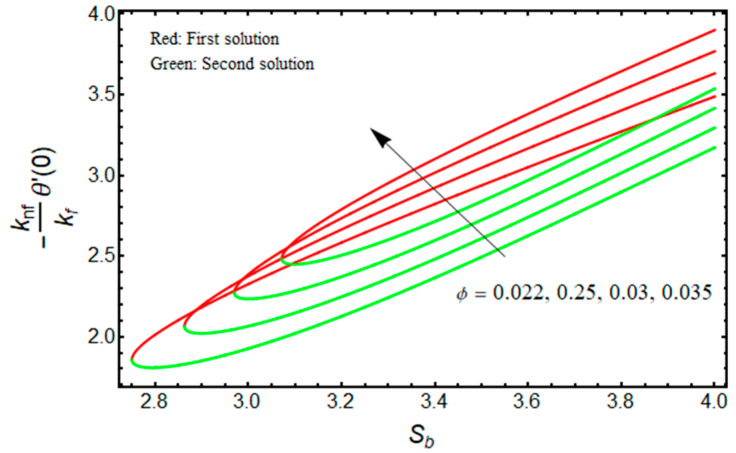
Effect of ϕ on $-\left(k_{nf}/k_{f}\right)\theta \prime \left(0\right)$ versus $S_{b}$ when $\chi =1,\;M_{b}=0.5,\;R_{d}=1.5,\;m=1$.

**Figure 12:**
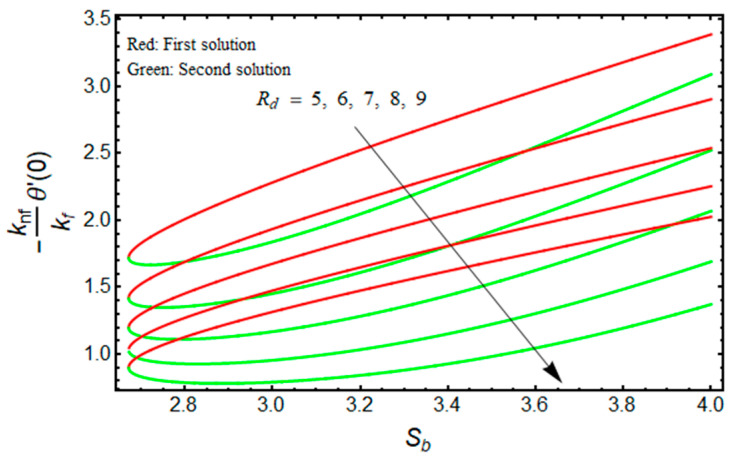
Effect of $R_{d}$ on $-\left(k_{\mathrm{nf}}/k_{f}\right)\theta \prime \left(0\right)$ versus $S_{b}$ when $\phi =0.025,\;\chi =1,\;M_{b}=0.5,\;m=1$.

**Figure 13:**
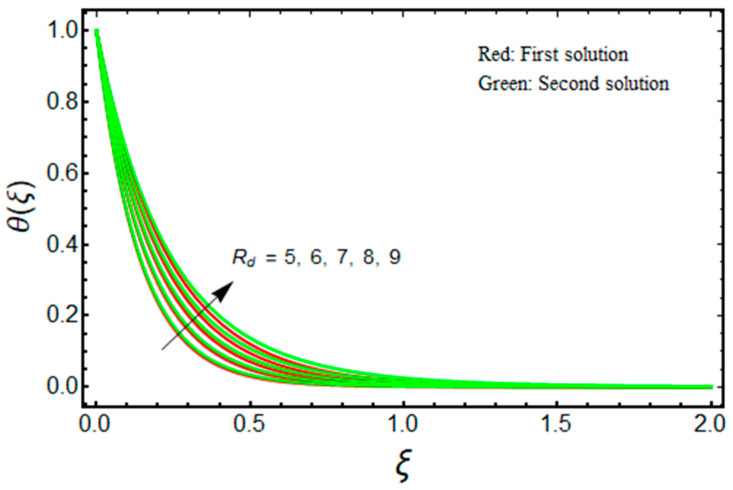
Effect of $R_{d}$ on θ(ξ) when $\phi =0.025,\;\chi =1,\;M_{b}=0.5,\;m=1$.

In this proposed portion, we essentially demonstrated, geometrically discussed, and physically interpreted the closed-form analytical solutions of the current water-Al_2_O_3_ nanofluid problem for the two distinct solution branches (first and second solutions), which may be shown visually in Figure 2, Figure 3, Figure 4, Figure 5, Figure 6, Figure 7, Figure 8, Figure 9, Figure 10, Figure 11, Figure 12 and Figure 13. In these prepared various distinct plots, we displayed the graphical behavior of the velocity profiles, temperature distribution, heat transfer, and wall drag force with the influence of various distinct factors, such as radiation parameter $R_{d}$, solid nanoparticle volume fractions ϕ, magnetic parameter $M_{b}$, mass suction parameter $S_{b}$, and non-Newtonian or Casson fluid parameter χ. The problem exhibited two distinct branch solutions (first and second), where the first branch solution in the entire paper is accessible by the solid red lines, and the second branch solution is revealed by the solid green lines. Moreover, the thermophysical properties of the aluminum oxide (Al_2_O_3_) nanoparticles and the base fluid (H_2_O) are arranged in Table 1, while the values of their constants of the water-Al_2_O_3_ nanofluid are written in Table 2. Table 3 and Table 4 are prepared to examine the suction effect on wall drag force and heat transfer. Table 3 indicates that the value of $\left(\mu_{nf}/\mu_{f}\right)G\prime \prime \left(0\right)$ increases for the outcome of the first branch and declines for the outcome of the second branch. This trend may imply a flow with separation. This trend may imply a flow with separation, in which the structure of a tiny wake may minimize the wall drag force past a shrinkable sheet. Thus, the values of $\left(\mu_{nf}/\mu_{f}\right)G\prime \prime \left(0\right)$ decline. Table 4 suggests the values of $-\left(k_{nf}/k_{f}\right)\theta \prime \left(0\right)$ augmenting due to greater intensity of suction in both branches of the solution. Physically, as the suction uplifts, it enhances the porosity of the sheet, which ultimately permits more nanoparticles to disperse the sheet. As a consequence, the rate of heat transfer is augmented as suction enhances.

Figure 2 and Figure 3 are organized to show the impact of $S_{b}$ on the velocity and temperature of the water-based aluminum oxide nanofluid for the two distinct branch solutions (first and second), respectively. The velocity upsurges with elevated values of $S_{b}$ for the first branch solution, while the contrary impact is observed for the branch of the second solution. Additionally, it is noticed from Figure 2 that the velocity boundary layer thickness (BLT) shrinks for the first branch solution and improves for the branch of the second solution due to the larger values of $S_{b}$. Alternatively, the temperature field declines significantly in both branches of the solution with the enrich values of $S_{b}$. Therefore, the thermal BLT reduces with $S_{b}$ for the first branch solution as well as for the second branch solution, which is graphically depicted in Figure 3. In addition, for the larger mass suction parameter temperature fields, overshoot is achieved merely in the branch of the second solution. About the physical observation, the stronger wall mass transfer parameter $S_{b}$ reduces the wall drag force to avoid the separations of the BL. The conclusion is that the field of velocity augments for the branch of the first solution, which is physically stable. Additionally, BLT shrinks with this continuous enhancement in the velocity and wall mass transfer parameter.

The impact of $M_{b}$ on the velocity and temperature of the water-based aluminum oxide nanofluid for the two distinct branch solutions (first and second) is portrayed in Figure 4 and Figure 5, respectively. It is noted that for improving the values of $M_{b}$, the fields of velocity magnitude and thermal (BLT) decrease in the branch of the first solution and become slightly thicker for the branch of the second solution. Moreover, the gap between the outcomes of the green curves is stronger and better when compared to the outcome of the red curves. Generally, the magnetic field powerfully distresses the nanoparticles; hence the magnitude of the velocity increases by increasing $M_{b}$, while the temperature profile is diminished.

The dimensionless velocity and temperature distribution for the first branch solution as well as for the second branch solution against ξ, with the significant impact of ϕ, is described in Figure 6 and Figure 7, respectively. It is noticed that the profile of velocity at a point decreases with ϕ for the first solution, and the influence of ϕ changes for the branch of the second outcome. However, the temperature field upsurges with ϕ for the first solution, and the impact of ϕ is similar for the second branch solution. Moreover, for the increasing values of ϕ, the velocity BLT is increased for the second solution and is decreased for the branch of the first solution, while the thermal BLT is significantly improved in both solution branches. Physically, the effects of the nanoparticles can disperse some energy in the compact form of heat. Simultaneously, if the influence of the aluminum oxide nanoparticle increases significantly, this may cause more dissipation of energy in the form of heat which yields to enhance thermal conductivity. As a result, the temperature profile and the thermal BLT are augmented for the first and second solution branches.

The quantities of $\left(\mu_{nf}/\mu_{f}\right)G\prime \prime \left(0\right)\;$ and $-\left(k_{nf}/k_{f}\right)\theta \prime \left(0\right)$ related to the friction factor (wall drag force) and heat transfer (wall temperature gradient) of the water-based aluminum oxide nanofluid for the first and second branch solutions versus $S_{b}$, owing to the effects of the Casson parameter χ, are graphically demonstrated in Figure 8 and Figure 9, respectively. Outcomes recommend that the values of $\left(\mu_{nf}/\mu_{f}\right)G\prime \prime \left(0\right)\;$ augments with the higher value of χ as well as the wall mass transfer parameter for the branch of the first solution, and it declines for the stronger Casson parameter χ for the branch of the second solution (see Figure 8) due to the elasticity of the fluid flow. Meanwhile, the wall temperature gradient $-\left(k_{nf}/k_{f}\right)\theta \prime \left(0\right)$ upsurges with χ for the branch of the first solution, but rises and falls in some specific portion of the domain (such as the wall mass transfer parameter) for the branch of the second solution (see Figure 9). In addition, the turning point of the existence of multiple solutions reduces with the larger values of $M_{b}$.

Figure 10 and Figure 11 illustrate the significant impact of ϕ on $\left(\mu_{nf}/\mu_{f}\right)G\prime \prime \left(0\right)\;$, and $-\left(k_{nf}/k_{f}\right)\theta \prime \left(0\right)$ against $S_{b}$ for the double solution branches, respectively. From Figure 10, it is deeply noticed that for increasing $S_{b}$ and ϕ, the friction factor is decreased for the branch of the first solution, and the impacts of ϕ and $S_{b}$ are upturned for the branch of the second solution. However, the heat transfer rate is uplifting for both solution branches with a higher value of ϕ (see Figure 11). Additionally, the turning points of the existence of solutions upsurge with stronger values of ϕ. This is due to the fact that nanofluid improves the temperature field in the boundary layer. The finding also acknowledges the physical behavior, as increasing the volume of nanoparticles augments the thermal conductivity. The thermophysical properties have a direct impact on the effectiveness of nanofluids in thermal systems.

Figure 12 depicts the behavior of $-\left(k_{nf}/k_{f}\right)\theta \prime \left(0\right)$ for $S_{b}$ as $R_{d}$ varies. The lower and upper branch solutions indicate the value of $-\left(k_{nf}/k_{f}\right)\theta \prime \left(0\right)$ decelerates as $R_{d}$ enhances. The stronger influence of radiation augments the nanofluid temperature inside the region of the boundary layer due to the fact that the conduction heat transport rate is augmented at the sheet surface. This fact is supported through the profile of temperature in Figure 13, where the nanofluid temperature uplifts when $R_{d}$ enhances. As a result, thermal conductivity rises, resulting in a decline in the convective heat transfer rate.

## 5. Conclusions

In the current exploration, the impact of magnetic fields on the radiative flow induced by Al_2_O_3_ nanofluid past a radially shrinking sheet was investigated. In addition, the effectiveness of fluid flow, viscosity, and thermal conductivity were examined using the KKL model. Closed-form double solutions of leading transport equations were presented. The effects of relevant parameters on the explained flow are discussed with the assistance of graphs. The important results of the investigations are gathered as follows:

- The suction and magnetic parameters accelerate the velocity in the first branch solution and decelerate in the second branch solution, whilst declining the temperature distribution in both branch solutions.
- The temperature profile uplifts due to the solid nanoparticle volume fraction in both solutions, while the velocity increases and decreases due to the solid nanoparticle volume fraction in the first and second branch solutions, respectively.
- The heat transfer and the friction factor increase due to the Casson parameter.
- The nanoparticle volume fraction augments the heat transfer and declines the friction factor.

The current findings highlight the importance of the technology of heat exchangers, storage of geothermal storage, materials processing, and all other procedures inspired by the concept of heat development. The utilization of heat transfer fluids incorporating the dispersion of nanoparticles to resolve the problems of cooling in thermal systems is a significant technological application.

## Figures and Tables

**Table 1:** Thermophysical properties of the nanofluid.

Physical Properties	Water	Al_2_O_3_
k (Wm^−1^K^−1^)	0.613	25
$c_{p}$ (J kg^−1^ K^−1^)	4179	765
ρ (kg m^−3^)	997.1	3970
$\sigma \;{\left(\Omega m\right)}^{-1}$	0.05	1 × 10^−10^
$d_{\mathrm{pa}}$ (nm)	-	47
Pr	6.2	-

**Table 2:** Constants of water-Al_2_O_3_ nanofluid.

Coefficient Values	Water-Al_2_O_3_
$c_{1}$	52.813
$c_{2}$	6.115
$c_{3}$	0.695
$c_{4}$	4.1 × 10^−2^
$c_{5}$	0.176
$c_{6}$	−2.98.198
$c_{7}$	−34.532
$c_{8}$	−3.922
$c_{9}$	−0.235
$c_{10}$	−0.999

**Table 3:** Values of $\left(\mu_{nf}/\mu_{f}\right)G\prime \prime \left(0\right)$ for distinct values of $S_{b},\chi ,\phi$, and $M_{b}$.

χ	$M_{b}$	ϕ	$S_{b}$	3.5	4	4.5	5
0.5	0.5	0.025	First solution	2.58605	3.40613	4.09306	4.73029
			Second solution	1.17470	0.89187	0.74219	0.64220
1	0.5	0.025	First solution	4.66415	5.63891	6.55804	7.44685
			Second solution	0.97697	0.80809	0.69483	0.61190
2	0.5	0.025	First solution	6.60110	7.81896	8.99505	10.14620
			Second solution	0.92040	0.77704	0.67544	0.59881
5	0.5	0.025	First solution	8.50938	9.98435	11.42330	12.83980
			Second solution	0.89249	0.76064	0.66483	0.59149
∞	0.5	0.025	First solution	10.4065	12.1435	13.8436	15.5307
			Second solution	0.87575	0.75048	0.65812	0.58680

**Table 4:** Values of $-\left(k_{nf}/k_{f}\right)\theta \prime \left(0\right)$ for distinct values of $S_{b},\chi ,\phi ,R_{d},m$, and $M_{b}$.

χ	$M_{b}$	ϕ	$R_{d}$	m	$S_{b}$	3.5	4	4.5	5
0.5	0.5	0.025	5	1	First solution	2.82016	3.52056	4.10507	4.66170
					Second solution	2.61293	3.32299	3.99296	4.68492
1	0.5	0.025	5	1	First solution	3.04693	3.63183	4.18821	4.72967
					Second solution	2.66077	3.44115	4.28216	5.18029
2	0.5	0.025	5	1	First solution	3.13405	3.69660	4.24086	4.77445
					Second solution	2.70750	3.72440	4.87577	6.24120
5	0.5	0.025	5	1	First solution	3.19054	3.74125	4.27820	4.80674
					Second solution	2.85264	4.31988	6.24757	9.17801
∞	0.5	0.025	5	1	First solution	3.23132	3.77438	4.30633	4.83129
					Second solution	3.18356	5.90969	11.6249	39.6040

## Data Availability

Not applicable.

## Nomenclature

$u_{w}\left(r_{b}\right)$	Velocity of the shrinking disk (m/s)
$B\left(r_{b}\right)$	Variable magnetic field strength (Tesla)
$B_{0}$	Constant magnetic field strength (Tesla)
$w_{w}\left(r_{b}\right)$	Wall mass transfer velocity (m/s)
$T_{w}\left(r_{b}\right)$	Wall temperature (K)
$v_{0},c$	Arbitrary constants
$T_{\infty }$	Ambient temperature (K)
$T_{b}$	Fluid temperature (K)
$q_{rad}$	Radiative heat flux
$T_{0}$	Constant reference temperature (K)
G(ξ)	Dimensionless velocity
$M_{b}$	Magnetic parameter
Pr	Prandtl number
$S_{b}$	Mass transfer factor
$Nu_{r_{b}}$	Heat transfer
$C_{g}$	Skin friction coefficient
Rerb	Reynolds number
D	Denotes the roots of the equation
G(ξ)	Dimensionless velocity
$R_{d}$	Radiation parameter
m	Relative factor of the power-law index
$k_{C}$	Mean absorption coefficient (1/m)
k	Thermal conductivity (W/(m·K))
$u_{b},w_{b}$	Velocity components along the *r_b_*- and *z_b_*-axes (m/s)
$\left(r_{b},\delta ,z_{b}\right)$	Cylindrical coordinates (m)
$c_{p}$	Specific heat at constant pressure (J/Kg·K)
$B_{1}$ and $B_{2}$	Arbitrary constants

## Greek Symbols

$\nu_{f}$	Kinematic viscosity (m^2^/s)
γ and h	Empirical functions
θ(ξ)	Dimensionless temperature
χ	Non-Newtonian or Casson fluid parameter
σ	Electrical conductivity (Ω^−1^m^−1^)
μ	Dynamic viscosity (N·s/m^2^)
ξ	Pseudo-similarity variable
ρ	Density (kg/m^3^)
$\sigma_{C}$	Stefan-Boltzmann constant (W/(m^2^·K^4^))
ψ	Stream function
η	New similarity variable
ϕ	Solid nanoparticle volume fraction

## Acronyms

KKL	Koo–Kleinstreuer–Li
MHD	Magnetohydrodynamics
EOF	Electro-osmotic forces
Al_2_O_3_	Alumina
2D, 3D	Two and three-dimensional
BCs	Boundary conditions
BLT	Boundary layer thickness
BLF	Boundary layer flow
VD	Viscous dissipation
TIR	Thermal interfacial resistance

## Subscripts

pa	Nanoparticles
nf	Nanofluid
f	Regular based-fluid
w	Wall boundary condition
∞	Far-field condition

## Superscript

‘	Derivative with respect to *ξ*

## Appendix A

The magnetic term in the balance of momentum (Equation (3)) is explained here. In the given paper, the magnetic body force, F, is defined by:

F=J×B

with the electric current vector, J:

$$J=\sigma_{nf}\left(E+V\times B\right)$$

where $\sigma_{nf}$ is the electrical conductivity of the nanofluid and E is the electric field vector. It is assumed that the imposed magnetic field, B=$\langle 0,0,r_{b}B_{0}\rangle$, acts in the $z_{b}-$direction, which is normal for the disk where the boundary layer is formed; the flow is confined to $z_{b}>0$. Based on the flow assumption due to a low Reynolds number, the induced magnetic field can be neglected. Moreover, the electric field vector, E, is assumed to be zero, i.e., the electric and polarization effects are neglected. Therefore,

$$\begin{array}{c} J=\sigma_{\mathrm{nf}}\left(V\times B\right)=\sigma_{\mathrm{nf}}\left[\langle u_{b},v_{b},w_{b}\rangle \times \langle 0,0,r_{b}B_{0}\rangle \right]\\ =\sigma_{\mathrm{nf}}\left|\begin{array}{ccc} i & j & k\\ u_{b} & v_{b} & w_{b}\\ 0 & 0 & r_{b}B_{0} \end{array}\right|\\ =\sigma_{\mathrm{nf}}\langle v_{b}r_{b}B_{0},-u_{b}r_{b}B_{0},0\rangle \end{array}$$

where $u_{b}$, $v_{b}$, and $w_{b}$ are the velocity components in the $r_{b}$, δ, and $z_{b}$ directions, respectively. Further, here in the considered problem, the velocity field, V, is assumed as $V=\langle u_{b},0,0\rangle$. Putting this in the above Equation (3), we get

$$J=\sigma_{nf}\left(V\times B\right)=\sigma_{nf}\langle 0,-u_{b}r_{b}B_{0},0\rangle$$

Then, the expression J×B can be written as:

$$\begin{array}{c} J\times B=\sigma_{\mathrm{nf}}\left[\langle 0,-u_{b}r_{b}B_{0},0\rangle \times \langle 0,0,r_{b}B_{0}\rangle \right],\\ =\sigma_{\mathrm{nf}}\left|\begin{array}{ccc} i & j & k\\ 0 & -u_{b}r_{b}B_{0} & 0\\ 0 & 0 & r_{b}B_{0} \end{array}\right|,\\ =\sigma_{\mathrm{nf}}\langle -u_{b}r_{b}{}^{2}B_{0}{}^{2},0,0\rangle . \end{array}$$

Thus,

$${\left(J\times B\right)}_{r_{b}}=-\sigma_{nf}r_{b}{}^{2}B_{0}{}^{2}u_{b},\;\;\;\;{\left(J\times B\right)}_{\delta }=0,\;\;\;\mathrm{and}\;\;\;{\left(J\times B\right)}_{z_{b}}=0,$$

where ${\left(J\times B\right)}_{r_{b}}$, ${\left(J\times B\right)}_{\delta }$, and ${\left(J\times B\right)}_{z_{b}}$ are the components of J×B in the $r_{b}$, δ, and $z_{b}$ directions, respectively. In addition, the expression of the electrical conductivity $\sigma_{nf}$ depends on ϕ.
